# Designing string-of-beads vaccines with optimal spacers

**DOI:** 10.1186/s13073-016-0263-6

**Published:** 2016-01-26

**Authors:** Benjamin Schubert, Oliver Kohlbacher

**Affiliations:** Center for Bioinformatics, University of Tübingen, 72076 Tübingen, Germany; Department of Computer Science, Applied Bioinformatics, 72076 Tübingen, Germany; Quantitative Biology Center, 72076 Tübingen, Germany; Faculty of Medicine, University of Tübingen, 72076 Tübingen, Germany

## Abstract

String-of-beads polypeptides allow convenient delivery of epitope-based vaccines. The success of a polypeptide relies on efficient processing: constituent epitopes need to be recovered while avoiding neo-epitopes from epitope junctions. Spacers between epitopes are employed to ensure this, but spacer selection is non-trivial.

We present a framework to determine optimally the length and sequence of a spacer through multi-objective optimization for human leukocyte antigen class I restricted polypeptides. The method yields string-of-bead vaccines with flexible spacer lengths that increase the predicted epitope recovery rate fivefold while reducing the immunogenicity from neo-epitopes by 44 % compared to designs without spacers.

**Electronic supplementary material:**

The online version of this article (doi:10.1186/s13073-016-0263-6) contains supplementary material, which is available to authorized users.

## Background

One of the most promising approaches of rational vaccine design uses so-called epitope-based vaccines (EVs). Vaccines based on T-cell epitopes, short immunogenic peptide sequences derived from antigens, offer several advantages over traditional whole attenuated or subunit vaccines [1]. Unlike traditional vaccines, EVs do not contain potentially infectious material and the selection of peptides can be tailored to address the genetic variation of pathogens and that of a target population or of an individual patient. Well-established techniques for peptide synthesis guarantee rapid high-quality production and an economical storage of the final vaccine [1].

Rational development of EVs relies on bioinformatics for prediction of viable epitopes. Machine-learning methods, such as probabilistic models, neural networks, and support vectors machines, are routinely used with high accuracy for epitope prediction [2–5]. Different algorithms have been suggested as well for selecting an optimal set of epitopes for EV design, each emphasizing different aspects of EVs [6–10]. Among these approaches is OptiTope, a mathematical framework that relies on integer linear programming, which can easily be adapted to many different settings and types of EVs [8, 11].

**Fig. 1:**
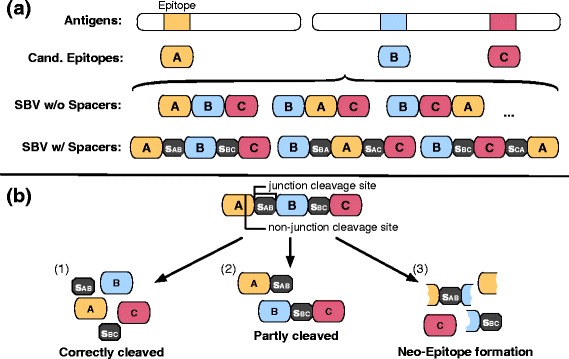
Rational string-of-beads design. **a** Design process of a string-of-beads vaccine (SBV). Given a set of antigen candidates, epitopes are derived either experimentally or computationally. A selection of *n* candidate epitopes is determined, which form the basis of the SBV. These epitopes are either directly combined into a polypeptide or small connecting sequences (spacers) are used to link adjacent epitopes. In total, there are *n*! possibilities to arrange *n* epitopes into a SBV. **b** Possible cleavage outcomes of a SBV. The efficacy of a SBV depends on correct proteasomal cleavage. Desired is a cleavage pattern that correctly recovers all contained epitopes *(1)*. Not all junction cleavage sites might be cleaved, which results in a partly cleaved and less effective SBV *(2)*. Cleavage of the SBV at non-junction sites can create neo-epitopes. Generation of neo-epitopes can induce unwanted immune responses and reduces the amount of desired epitopes generated by the SBV *(3)*

Nevertheless, the stability and delivery of EVs remain major obstacles. Several strategies have been explored in clinical studies and range from administration of peptide cocktails to assembly of selected peptides into polypeptides [12]. One popular approach concatenates the epitope sequences, like beads on a string, to create a string-of-beads vaccine (SBV, Fig. 1a). The efficacy of an SBV depends on the processing of the polypeptide such that the majority of desired T-cell epitopes are recovered and subsequently presented by human leucocyte antigen (HLA) molecules. A major factor for optimal recovery is the correct cleavage of the epitopes. It has been shown that recovery of the epitopes is strongly linked to the ordering of the peptides within the SBV due to its influence on the cleavage probability [13]. An unfavorable order can lead to miscleaved peptides and thus, to an ineffective vaccine (Fig. 1b). Furthermore, new cleavage sites and neo-epitopes can arise from non-native sequences at junctions between epitopes and/or spacers. These neo-epitopes can also have detrimental effects [14] (Fig. 1b).

To improve the recovery of epitopes in SBVs, several groups have suggested the use of spacer sequences between epitopes [15–17] (Fig. 1a). However, it is unclear how to determine the optimal length and amino acid sequence of a spacer to exploit fully its potential. Furthermore, with increasing spacer length, the problem of induced neo-epitopes and new arising cleavage sites becomes increasingly challenging. In addition, experimentally testing designs to determine an optimal SBV, even without considering spacer sequences, quickly becomes infeasible. A dozen epitopes can be combined into about half a billion (12!) distinct SBV sequences. Considering additional spacer sequences with flexible length, increases the possibilities many times over. For instance, allowing spacer sequences up to a length of three for 12 epitopes results in over 44 trillion possible designs. For simplicity, most SBV designs have so far used fixed spacer sequences. Until now, only a few computational approaches have been proposed to address the epitope assembly problem (i.e., the problem of choosing the right epitope order). Vider-Shalit et al*.* suggested a genetic algorithm that simultaneously performs epitope selection and assembly [6]. Toussaint et al. reduced the epitope assembly problem to the well-known traveling salesperson problem (TSP) and solved it heuristically or optimally via integer linear programming [7]. Neither of these approaches considers spacer sequences though.

In this work, we propose an approach to determine a provably optimal spacer sequence of fixed length for a given HLA-I restricted epitope pair. We also extend the formulation to determine the optimal spacer length and combine this approach with that of Toussaint et al. [7] to design an optimal SBV with flexible spacer sequences. Additionally, we account for the problem of arising neo-epitopes and cleavage sites by formulating the problem of designing a spacer sequence as a multi-objective optimization problem that maximizes the recovery probability of the desired epitopes, minimizes the immunogenicity of neo-epitopes, and (optionally) minimizes the cleavage probability at non-junction sites at the same time. We focus our efforts solely on HLA-I antigen processing, since computational prediction methods for proteasomal cleavage and HLA-I binding are well established. The cleavage-site prediction models are used for designing spacer sequences and for ordering the therapeutic epitopes of the SBV to increase their cleavage likelihood artificially, whereas the HLA-I binding prediction models are used to hinder the formation of neo-epitopes at the epitope–spacer interfaces. Note that an experimental determination of such an optimal design is virtually impossible due to the vast number of possible designs; a computational approach is, thus, indispensable.

Our results indicate there is a strong increase in the number of correctly cleaved epitopes and a decrease in the neo-immunogenicity of the complete construct compared to SBV designs with commonly used fixed spacers and optimally arranged SBVs without spacer sequences.

## Methods

### Optimization problem from an immunological perspective

The goal of the optimization is to design a SBV based on a given set of *N* epitopes. The SBV construct will contain all epitopes, but the ordering of the epitopes, as well as the length and sequence of the *N* – 1 spacers between these epitopes, is variable. The SBV is designed in a way that (a) maximizes the recovery of the epitopes while (b) minimizing the production of undesired neo-epitopes.

More formally: Given a set *E* of *N* epitopes *e*_1,_ …, *e*_*N*_, we specify an optimal spacer *s*_*ij*_ of length *k* defined over the alphabet of amino acids *Σ* that connects two epitopes ${e}_i\in {\Sigma}^{\left|{e}_i\right|}$ and ${e}_j\in {\Sigma}^{\left|{e}_j\right|}$ as the sequence that maximizes the likelihood of it being cleaved at the respective junction cleavage sites *c*_*i*_ and *c*_*j*_ of the two epitopes. This increases the likelihood of recovering all desired epitopes (Fig. 1b), which in turn increases the likelihood of them being loaded and presented on HLA-I molecules. If only a few epitopes are correctly processed and neo-epitopes are formed (Fig. 1b), the influence of these neo-epitopes on the immunological processes should be minimized, so that the risk of undesired immune responses is reduced. This can be achieved by designing the spacer sequences in such a way that the potential neo-epitopes spanning the connected epitopes *e*_*i*_, *e*_*j*_ and their spacer *s*_*ij*_ are minimally immunogenic. To approach this problem computationally, proteasomal cleavage and immunogenicity prediction models are needed. In T-cell epitope prediction, proteasomal cleavage prediction was found to have a minor impact on prediction performance [18, 19]. However, in the context of in silico string-of-beads design, its impact is much more pronounced. Here, accurate cleavage prediction is important for predicting the recovery probabilities of the desired epitopes of the SBV, maximizing the individual cleavage probability by rearranging the order of the epitopes, and optimizing spacer sequences. These effects have been shown to be essential for a vaccine’s efficacy in several experimental studies [13–16].

In the following, we describe the prediction models used and derive the mathematical formulation to tackle the problem of designing a SBV with flexible spacer sequences. It should be mentioned that the developed framework is restricted to linear prediction methods. Non-linear prediction models, like artificial neural networks (e.g., NetMHC [3]), or even more complex prediction approaches like the one proposed by Zhang et al. [19], would lead to a non-convex, non-linear mixed integer optimization problem that cannot be solved efficiently and optimally even for small instances [20]. Furthermore, the linear prediction methods have to be fully integrated into the optimization framework to be able to solve the corresponding optimization problem efficiently. Integrated linear methods for epitope and cleavage prediction are listed in “Implementation”.

### Cleavage site model

For cleavage site prediction, we employ the position-specific scoring matrix (PSSM) *ϕ*_*C*_(∙) proposed by Dönnes et al., which uses four C-terminal amino acids and two N-terminal amino acids to predict a cleavage site. It has been shown to give quite robust and generalizable predictions [18].

We define the cleavage objective of spacer *s*_*ij*_ and epitope pair *e*_*i*_, *e*_*j*_ as the linear combination of the individual cleavage likelihoods of site *c*_*i*_ and *c*_*j*_ predicted by the PSSM *ϕ*_*C*_:

1 $$ C\left({e}_i,{e}_j\Big|{s}_{ij}\right):={\displaystyle \sum_{l=0}^{n_c-1}}{\phi}_C\left(S\left[{i}_c+l\right],l\right)+{\phi}_C\left(S\left[{j}_c+l\right],l\right). $$

Here *S* ∶ = *e*_*i*_*s*_*ij*_*e*_*j*_ denotes the concatenated sequence of a spacer and its enclosing epitope pair *e*_*i*_ and *e*_*j*_. *S*[*x*] indicates the *x*th character of sequence *S*, *n*_*c*_ represents the number of amino acids used to predict a cleavage site, and *i*_*c*_, *j*_*c*_ denote the start of the segments used to predict the cleavage likelihoods at site *c*_*i*_ and *c*_*j*_, respectively. The PSSM *ϕ*_*C*_ is a 20 × *n*_*c*_ matrix, where each row represents an amino acid and each column the position within a sequence of length *n*_*c*_. The entry *ϕ*_*C*_(*a*, *i*) of an amino acid *a* at position *i* represents the influence of an amino acid at a particular position on the cleavage likelihood. Thus, the log-likelihood of being cleaved is obtained by summing over the entries of *ϕ*_*C*_ for a given sequence of length *n*_*c*_.

### Immunogenicity model

Our immunogenicity model is based on the formulation proposed by Toussaint et al*.*, which assumes that each epitope independently influences the immune response with respect to a target population or individual represented by a set of HLA alleles *H* [8]. The contribution of an HLA allele *h* ∈ *H* is directly proportional to the probability *p*_*h*_ of the allele occurring within any patient of the target population *H*. We, thus, obtain

2 $$ I\left(S\Big|H\right):=\sum_{h\in H}{p}_h\sum_{i=1}^{n-{n}_e} \max \left(0,\left(\sum_{j=0}^{n_e-1}{\phi}_I\left(h,S\left[i+j\right],j\right)\right)-{\tau}_h\right) $$

where *S* is the input sequence of length *n. ϕ*_*I*_(∙) represents a linear model predicting the immunogenicity of an epitope of length *n*_*e*_ for an HLA allele *h* ∈ *H* and *τ*_*h*_ characterizes the threshold of the HLA allele. For the immunogenicity predictor, we use SYFPEITHI, a PSSM generated from natural processed HLA ligands [2].

### Problem definition as multi-objective optimization

From the discussion of the previous sections, it becomes apparent that for successfully designing a spacer sequence *s*_*ij*_ for an epitope pair *e*_*i*_, *e*_*j*_, one has to consider multiple design goals. On the one hand, the spacer sequence should be designed to maximize the cleavage probabilities of the cleavage sites *c*_*i*_ and *c*_*j*_. On the other hand, it should also minimize the neo-immunogenicity *I*(∙) of the complete sequence *S* := *e*_*i*_*s*_*ij*_*e*_*j*_. Such problems can be conveniently described as multi-objective optimization problems. Solving a multi-objective optimization problem yields Pareto-optimal solutions that resemble trade-offs between all objective functions.

Most approaches for solving multi-objective optimization problems use scalarization techniques combining the different objectives [21]. A common approach linearly combines the objectives weighted by a coefficient reflecting the designers’ preferences. However, identifying the best weights is difficult because (a) the numerical properties of the objective functions usually differ and (b) the effect of the defined weights is hard to determine a priori.

Since our stated problem exhibits a clear ordering of the objectives with respect to their priority, namely junction-cleavage likelihood over neo-immunogenicity, the problem of finding a Pareto-optimal solution can be significantly simplified by applying lexicographical ordered optimization (LO). In LO, the objectives are ordered based on their importance and several single objective problems of the following form are iteratively solved:

3 $$ \begin{array}{l}\underset{x}{ \min }{f}_i(x)\\ {}\mathrm{s}.\mathrm{t}.\kern0.28em {f}_j(x)\le {f}_j\left({x}^{\ast}\right)\\ {}\mathrm{where}\kern0.28em i\in \left\{1,N\right\},\kern0.28em j\in \left\{1,i-1\right\}\kern0.28em \mathrm{if}\kern0.28em i>1,\end{array} $$

where *i* represents the priority of the objective function, and *f*_*j*_(*x*_j_^*^) the optimum of the *j*th objective function found at the *j*th iteration [22]. Note that after the first iteration, *f*_*j*_(*x*_j_^*^) does not necessarily obtain the same solution as the independent optimization of *f*_*j*_(*x*), since new constraints have been added to the problem formulation.

### Spacer design with fixed length

We now formulate the problem of designing a spacer of fixed length *k* as a bi-objective mixed integer linear program (ILP). We represent each position *i* and amino acid *a* of the concatenated sequence of spacer and epitope pairs with a binary decision variable *x*_*i*,*a*_. Additionally, we allow all 20 amino acids to appear within the spacer sequence. A constraint has to be added to allow only one amino acid per position. The complete Pareto formulation has, thus, the following form:

4 $$ \begin{array}{l}\underset{x}{ \max}\sum_{l=0}^{n_c-1}\left(\sum_{a\in {S}_{i_c+l}}{x}_{i_c+l,a}{\phi}_C\left(a,l\right)+\sum_{b\in {S}_{j_c+l}}{x}_{j_c+l,b}{\phi}_C\left(b,l\right)\right)\\ {}\underset{x}{ \min}\sum_{h\in H}{p}_h\sum_{i=1}^{n-{n}_e} \max \left(0,\left(\sum_{j=0}^{n_e-1}\sum_{a\in {S}_{i+j}}{\mathrm{x}}_{i+j,a}{\phi}_{\mathrm{I}}\left(h,a,j\right)\right)-{\tau}_h\right)\\ {}\mathrm{s}.\mathrm{t}.{\displaystyle \sum_{a\in {S}_i}}{x}_{i,a}\le 1,\kern0.75em \forall i\in \left\{1,n\right\},\end{array} $$

where *S*_*i*_ denotes the set of amino acids allowed at position *i*.

Following the LO definition, we solve two consecutive ILPs to yield a lexicographically optimal solution:

$$ \begin{array}{l}{\mathrm{LO}}_{\mathrm{spacer}}\left({e}_i,{e}_j,k\right)\ :=\\ {}\begin{array}{cc}\mathrm{P}1\hfill & \begin{array}{l}{z}_1^{*}:=\underset{x}{ \max }{\displaystyle \sum_{l=0}^{n_c-1}}\left({\displaystyle \sum_{a\in {S}_{i_c+l}}}{x}_{i_c+l,a}{\phi}_C\left(a,l\right)+{\displaystyle \sum_{b\in {S}_{j_c+l}}}{x}_{j_c+l,b}{\phi}_C\left(b,l\right)\right)\\ {}\mathrm{s}.\mathrm{t}.\kern1em {\displaystyle \sum_{a\in {S}_i}}{x}_{i,a}\le 1, \kern0.5em \forall\ i\in \left\{1,n\right\}\end{array}\hfill \end{array}\\ {}\begin{array}{cc}\hfill \mathrm{P}2\hfill & \hfill \begin{array}{l}{z}_2^{*}: = \underset{x}{ \min }{\displaystyle \sum_{h\in H}}{p}_h{\displaystyle \sum_{i=1}^{n-{n}_e}} \max \left(0,\left({\displaystyle \sum_{j=0}^{n_e-1}}{\displaystyle \sum_{a\in {S}_{i+j}}}{x}_{i+j,a}{\phi}_I\left(h,a,j\right)\right)-{\tau}_h\right)\\ {}\mathrm{s}.\mathrm{t}.\kern1em {\displaystyle \sum_{a\in {S}_i}}{x}_{i,a}\le 1,\kern0.75em \forall\ i\in \left\{1,n\right\}\\ {}{\displaystyle \sum_{l=0}^{n_c-1}}\left({\displaystyle \sum_{a\in {S}_{i_c+l}}}{x}_{i_c+l,a}{\phi}_C\left(a,l\right)+{\displaystyle \sum_{b\in {S}_{j_c+l}}}{x}_{j_c+l,b}{\phi}_C\left(b,l\right)\right)\ge \alpha {z}_1^{*}\end{array}\hfill \end{array}\end{array} $$

Here, we restrict P2 to obtain at least *α* ∈ [0, 1] fraction of the maximal cleavage score achieved by solving P1. *α* represents the trade-off between cleavage likelihood and the likelihood of decreasing the immunogenicity score.

### String-of-beads design with spacers of flexible length

**Fig. 2:**
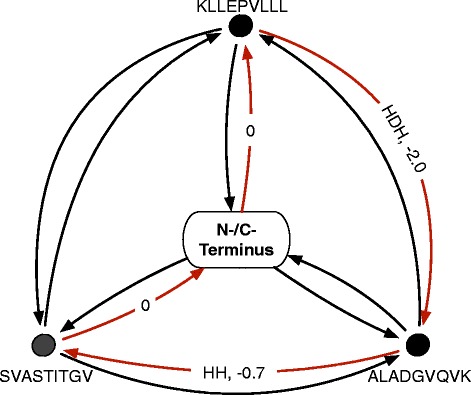
Example of a string-of-beads traveling salesperson (TSP) graph. Solving a TSP yields the shortest round trip, which visits each node exactly once. To solve the epitope assembly problem, each epitope is assigned to a node and artificial start and end nodes, representing the N- and C-terminals of the SBV, are added to the graph. The edges are weighted by the negative cleavage likelihood ratios of the two adjacent epitopes and labeled with the corresponding spacer of the epitope pair. *Red edges* mark the optimal round trip leading to an SBV of KLLEEVLLL-HDH-ALADGVQKV-HH-SVASTTTGV

To design a complete string-of-beads with flexible spacer lengths, the introduced LO formulation is iteratively solved for each pair *e*_*i*_, *e*_*j*_ ∈ *E* of epitopes with varying spacer length *k* ∈ {0, …, *K*}. The design with the highest minimum of both cleavage site likelihoods is selected and the scores obtained are used to initialize a fully connected and directed graph, where the negative cleavage scores represent the weights of the edges between epitopes pairs. Following Toussaint et al*.*, a TSP instance is formulated based on this graph by adding a node that represents the N- and C-termini of the SBV and connecting it with all other nodes with zero edge weights (Fig. 2). Solving this formulated TSP instance yields an optimal ordering of the epitopes. Together with the optimized spacers, we thus, obtain an optimal sequence for the entire vaccine construct. The description of the algorithm in pseudo-code can be found in Additional file 1.

### Non-junction cleavage site minimization

Besides the maximization of the junction cleavage likelihood, minimizing the likelihood of being cleaved at any other position will also improve the recovery probability of the therapeutic epitopes. Non-junction cleavage sites are partly influenced by the length of the spacer sequence and the epitope pairing. Therefore, we treat the minimization of non-junction cleavage sites as an optional third design goal and add to the sequence of consecutively solved ILPs a third optimization problem of the form:

$$ \begin{array}{l}{\mathrm{LO}}_{\mathrm{spacerEx}}\left({e}_i,{e}_j,k\right):=\dots \\ {}\begin{array}{cc}\kern1em \mathrm{P}3\kern1em & \kern1em \begin{array}{l}\underset{x}{ \min}\sum_{i=1}^{n-{n}_c}\sum_{j=0}^{n_c-1}\sum_{a\in {S}_{i+j}}{x}_{i+j,a}{\phi}_C\left(a,j\right)\\ {}\mathrm{s}.\mathrm{t}.\sum_{a\in {S}_i}{x}_{i,a}\le 1,\kern0.75em \forall i\in \left\{1,n\right\}\\ {}\sum_{l=0}^{n_c-1}\left(\sum_{a\in {S}_{i_c+l}}{x}_{i_c+l,a}{\phi}_C\left(a,l\right)+\sum_{b\in {S}_{j_c+l}}{x}_{j_c+l,b}{\phi}_C\left(b,l\right)\right)\ge \alpha {z}_1^{\ast}\\ {}\sum_{h\in H}{p}_h\sum_{i=1}^{n-{n}_e} \max \left(0,\left(\sum_{\mathrm{j}=0}^{{\mathrm{n}}_{\mathrm{e}}-1}\sum_{a\in {S}_{i+j}}{x}_{i+j,a}{\phi}_{\mathrm{I}}\left(h,a,j\right)\right)-{\tau}_h\right)\le \left(2-\beta \right){z}_2^{\ast}\end{array}\kern1em \end{array}\end{array} $$

Here again, *α* and *β* represent the trade-offs between the three objective functions. The influence of *α* and *β* on cleavage likelihood, neo-immunogenicity, and non-junction cleavage likelihood is depicted in Additional file 2.

### Implementation

To solve the problem efficiently, the spacer design was parallelized and the TSP solution was approximated using the Lin–Kernighan–Helsgaun heuristic [23]. The model was implemented in Python 2.7 using Pyomo 4.0 [24] and solved with ILOG CPLEX 12.5 (www.ilog.com) and the Lin–Kernighan–Helsgaun heuristic [23]. The complete framework was integrated into EpiToolKit, a web-based platform for rational vaccine design. It can be accessed at www.epitoolkit.de under *Spacer Design* [25]. The source code and example files can be found at https://github.com/FRED-2/OptiVac. The implementations currently support SYFPEITHI [2], BIMAS [26], SMM [27], and SMMPMBEC [28] for epitope prediction, and PCM [18] and ProteaSMM [29] for proteasomal cleavage prediction. The statistical analysis was conducted using R (www.r-project.org). Statistical significance was considered at a significance level of 0.05. Data used in the statistical analysis can be found in Additional files 3 and 4.

## Results

### Designed spacers increase cleavage likelihood and decrease neo-immunogenicity

**Fig. 3:**
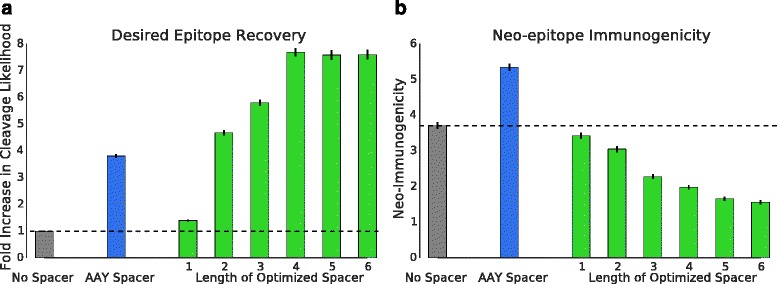
Fold change in cleavage likelihood and differences in neo-immunogenicity compared for 1000 randomly sampled epitope pairs. Spacers of lengths 1–6 were designed with the described model. The cleavage probability (**a**) and immunogenicity (**b**) were compared for epitope pairs concatenated without a spacer sequence, epitope pairs combined with a commonly used spacer sequence (AAY), and pairs combined with optimally designed spacers. *Black error bars* represent the 68 % confidence intervals

To validate the model performance, 1000 random epitope pairs, predicted for proteins of the cytomegalic virus strain AD169 (UniProt Proteom ID UP000008991), were generated and spacers of length 1–6 designed and optimized for the HLA distribution of the European population using *α* = 0.99. The fold change in cleavage likelihood as well as neo-immunogenicity were compared for concatenated epitopes without spacers, a commonly used fixed spacer (AAY) [16, 30, 31], and with optimally determined spacers (Fig. 3).

For each spacer length, a significant increase in cleavage likelihood could be observed for epitope pairs with optimized spacers compared to epitope pairs without spacers (paired one-sided Wilcoxon rank-sum test, Bonferroni corrected). In addition, the optimized spacers outperformed the constructs with a fixed spacer after a length of two (paired one-sided Wilcoxon rank-sum test, Bonferroni corrected). The maximum increase in cleavage likelihood was achieved with a spacer length of four, which is not surprising since the applied cleavage model uses four C- and two N-terminal amino acids to predict a cleavage site. The use of optimal spacer sequences resulted in a 7.7-fold increase in cleavage likelihood compared to epitope pairs without spacer sequences and a twofold increase compared to epitope pairs with a fixed AAY spacer.

In addition, significant improvements could be observed in terms of reduced neo-immunogenicity when using optimized spacers compared to both designs with fixed spacers and without spacers (paired one-sided Wilcoxon rank-sum test, Bonferroni corrected). With increasing spacer length, the immunogenicity decreased when using optimal spacer sequences. An average neo-immunogenicity reduction of 1.9-fold and 2.7-fold could be achieved at a spacer length of four compared to epitope pairs without spacers and fixed spacers, respectively. Detailed results can be found in Additional file 3.

### String-of-beads designs with optimal spacers improve epitope recovery

**Fig. 4:**
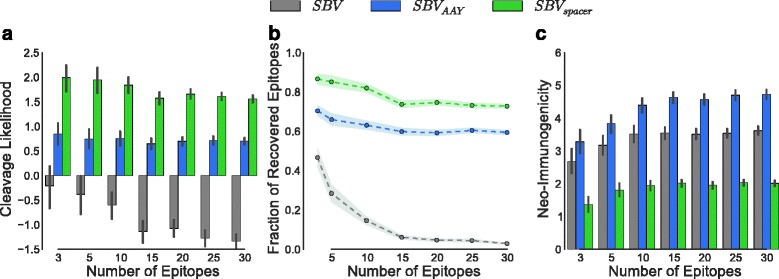
Comparison of string-of-beads with and without spacer sequences. Average junction cleavage likelihood (**a**), recovery percentage (**b**), and neo-immunogenicity (**c**) were measured for optimal string-of-beads designs with, without, and fixed AAY spacers. The string-of-beads constructs comprised three to 30 randomly selected epitopes. For each set size, the sampling was repeated 50 times. The maximum spacer length was set to *k* = 6. *Black error bars* and *colored outlines* represent the 68 % confidence intervals

A pool of epitopes was produced. The epitopes were predicted to bind to at least one HLA allele present in a European population. Out of this pool, random sets of size *l* ∈ {3, 5, 10, 15, 20, 25, 30} were selected. The optimal ordering was determined for the string-of-beads construct without (SBV) and with spacer sequences (SBV_spacer_) for a maximum spacer length of *k* = 6 amino acids. Additionally, ten randomly ordered strings-of-beads with fixed AAY spacers (SBV_AAY_) for the given epitope set were generated. This procedure was repeated 50 times for each set size. The junction cleavage likelihood averaged over the number of arising junction sites, the fraction of recovered epitopes (i.e., epitopes with preceding and succeeding C-terminal cleavage sites with positive cleavage score), as well as the neo-immunogenicity of the complete construct normalized by the number of included epitopes were compared between the strings-of-beads with a spacer, without spacer sequences, and the average performance of the random constructs with fixed spacers (Fig. 4).

The average junction cleavage scores of SBV_spacer_ and SBV_AAY_ were stable and well above the cleavage threshold of 0.0 for all set sizes, with an average score of 1.74 ± 0.63 and 0.73 ± 0.53, respectively. The average junction cleavage score for SBV decreased with increasing set sizes and was below the cleavage threshold even for small set sizes with an average score of −0.85 ± 1.09. This was also reflected in the percentage of recovered epitopes. SBV exhibited a decreasing recovery with increasing set sizes with an average of 15.4 ± 24.3 %, while SBV_spacer_ and SBV_AAY_ achieved a stable average recovery of 78.3 ± 16.2 % and 62.7 ± 15.2 % corresponding to a fivefold and fourfold increase, respectively. SBV_spacer_ also consistently outperformed SBV_AAY_, both in cleavage likelihood (2.38-fold increase) and recovery rate (1.25-fold increase).

The differences in neo-immunogenicity were not as strong, which is expected due to the chosen value of *α.* SBV_spacer_ consistently achieved a lower neo-immunogenicity score (average 1.88 ± 0.59) than SBV (average 3.37 ± 0.93) and SBV_AAY_ (average 4.31 ± 0.99), resulting in a decrease of 44.2 % and 56.8 %, respectively.

The optimal spacer length averaged at 3.23 ± 0.50 amino acids. The run time for instances with 30 epitopes was 5 min on average (maximum 5.6 min) on current commodity hardware (12-core Intel Xeon E5-2620 running at 2 GHz). Detailed results can be found in Additional file 4.

### Commonly used spacer designs tend to be worse than optimal designs

Several spacer sequences have been proposed in various settings ranging from a prophylactic vaccine to therapeutic cancer vaccine studies [15, 16, 30, 32–34]. However, these spacer sequences are not universally applicable and their usefulness is dependent on the epitope pairs they connect. To show the potential efficacy of the proposed model, we compared multiepitope studies that used spacers with our in silico designed spacers in terms of epitope recovery and induced neo-epitopes. An epitope was considered recovered if its preceding and succeeding cleavage sites were likely to be cleaved, as predicted by PCM (i.e. PCM score > 0.0). Neo-epitope prediction was performed with SYFPEITHI using the default threshold (i.e. SYFPEITHI score ≥ 20). Additionally, we computed the optimal ordering and selection of the experimental spacers similar to the approach in [35].

**Fig. 5:**
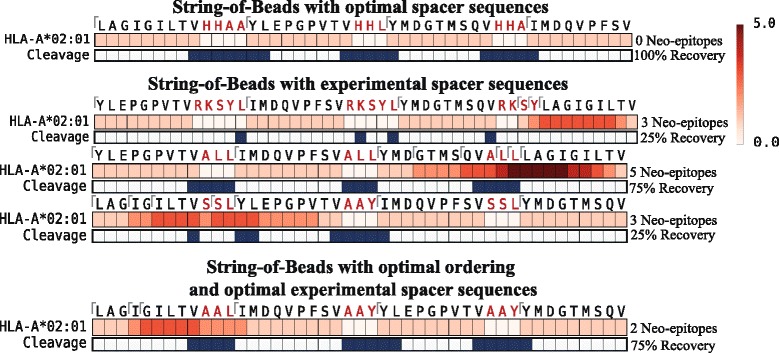
Comparison between experimentally used spacer sequences and in silico designed spacer sequences for the multiepitope polypeptide proposed by Levy et al. *Red bars* represent predicted epitopes and the intensity indicates overlapping epitopes at that position. The *blue rectangles* represent predicted C-terminal cleavage sites. Spacer sequences are marked in *red*. A *tick* indicates the start position of a predicted nine-mer epitope. Epitope and cleavage site predictions were performed with SYFPEITHI and PCM, respectively. A peptide was predicted as an epitope if its prediction score was equal to or above a threshold of 20 (default threshold of SYFPEITHI). A cleavage site was said to be cleaved if the predicted PCM score was above zero. An epitope was defined as recovered if both preceding and succeeding cleavage sites were predicted to be cleaved

Levy et al*.* proposed a therapeutic multiepitope polypeptide consisting of HLA-A*02:01 restricted modified epitopes derived from different melanoma-associated antigens (gp100:209–217(210 M): IMDQVPFSV, gp100:280–288(288 V): YLEPGEVTV; Mart1:27–35(27 L): LAGIGILTV; tyrosinase: 368–376(370D): YMDGTMSQV) and showed the proteasomal-dependent efficacy in vitro using the peripheral blood mononuclear cells of healthy donors and patients undergoing treatment [30]. To combine the selected peptides, a natively derived spacer sequence (RKSY(L)) as well as experimentally derived spacers (AAY and ALL/SSL) were used. The selected epitopes were included multiple times in the polypeptide combined with the different spacers to maximize the recovery probability. Therefore, we compared the different segments of the vaccine that were connected with the same spacer sequences (Fig. 5). Detailed results of the neo-epitope and cleavage site predictions can be found in Additional file 5.

In general, the optimal SBV design outperformed the experimentally used spacer sequences both in terms of therapeutic epitope recovery and in reduced neo-epitope appearance. With the designed spacers, 100 % of therapeutic epitopes could be recovered without generating neo-epitopes spanning the spacer sequences. The experimentally used spacers, on the other hand, either generated neo-epitopes or were not able to recover an essential amount of the therapeutic epitopes. With the spacer RKSY(L), only one out of four epitopes could be recovered, and ALL induced five neo-epitopes spanning the spacer. The Mart1-derived epitope and the combination of SLL and AAY generated neo-epitopes and resulted in the recovery of one out of four epitopes only. Even the design with optimally ordered epitopes and selected experimental spacer sequences could not recover all epitopes and introduced neo-epitopes. To establish the effect of different (linear) epitope prediction methods, the comparison was repeated with different methods (BIMAS [26] and SMM [27]). The recovery analysis was again performed with PCM, and default thresholds for BIMAS (predicted *T*_1/2_ ≥ 100) and SMM (predicted IC_50_ ≤ 500 nM) were used for neo-epitope detection. All therapeutic epitopes could be recovered using the in silico designed spacers with a smaller or equal number of neo-epitopes compared to the best experimentally used spacer sequence. While there are differences in detail between the methods, their overall behavior remained the same. Differences can be attributed to variations in the prediction accuracy of the methods (Additional file 5 and 6).

Similar results could be observed for the SBV construct proposed by Ding et al*.* [15] (Additional files 7 and 8). The proposed SBV was composed of T-cell epitopes derived from the hepatitis B virus X protein, which were combined with different spacer sequences to reduce the number of junction neo-epitopes. With the in silico designed spacer sequences, all therapeutic epitopes could be recovered without introducing neo-epitopes, whereas the experimentally used spacers induced neo-epitopes and were not able to recover all therapeutic epitopes.

## Conclusion

In this work, we propose a mathematical model for designing spacer sequences of flexible length for SBVs by exploiting existing proteasomal cleavage and epitope prediction methods. We combined the model with a TSP approach for optimal epitope ordering. We also addressed the problem of neo-epitopes and non-junction cleavage sites arising from spacer sequences and the order of the epitopes within the string-of-beads by extending the formulation with two additional objective functions. To solve the multi-objective optimization problem efficiently, we employ lexicographical optimization techniques.

The efficacy of the model was shown by comparing the recovery rates and neo-immunogenicity of optimal designs with commonly used fixed spacer sequences and spacer-less designs. In each case, the optimal design led to increased predicted epitope recovery and reduced generation of neo-antigens.

We also compared experimentally tested string-of-beads designs that used spacer sequences with our optimized designs. The experimentally used spacer sequences were often sub-optimally chosen for the connecting epitopes. As a consequence, there were neo-epitopes spanning the spacer sequences or proteasomal cleavage could not be guided to cleave the therapeutic epitopes correctly. In contrast, the in silico designed string-of-beads with optimally determined spacers showed improved cleavage patterns and reduced neo-immunogenicity. Often all therapeutic epitopes could be correctly cleaved without introducing neo-epitopes.

An obvious limitation of the current method is its reliance on computational models for proteasomal cleavage and epitope prediction. While models for HLA class I binding prediction exhibit a high accuracy, proteasomal cleavage models still leave room for improvements [36]. Currently, the approach is restricted to HLA class I epitopes but could be effortlessly extended once a cleavage prediction method for HLA-II ligands becomes available. In addition, the framework is designed flexibly enough to replace the underlying proteasomal cleavage prediction method, once more reliable computational prediction models are published. An experimental validation of selected optimal spacer designs is a non-trivial task. It cannot be performed as exhaustively as our computational study – the number of possible designs is simply too large. An experimental validation will thus, most likely, be limited to comparing only a few selected optimal designs to fixed spacer or spacer-less designs. Such validation is planned as future work together with experimental partners.

In conclusion, our method is a first framework that optimally designs both epitope order and spacers for SBV design. The mathematical method employs state-of-the-art prediction methods, but does not depend on specific methods. Our model predicts an increased recovery of desired epitopes and a reduced production of neo-epitopes compared to both fixed spacer and spacer-less designs.

## Additional files

Additional file 1:
**Algorithm for string-of-beads design with flexible spacer sequences.** A description in pseudo-code of the algorithm to determine the optimal ordering of epitopes and spacers for a string-of-beads vaccine. (PDF 1056 kb) Additional file 2:
**Influence of α and β on cleavage likelihood, neo-immunogenicity, and non-junction cleavage likelihood exemplified for spacers of length three.** Cleavage likelihood and neo-immunogenicity decrease linearly with *α*. For the conservatively chosen *α* = 0.99, *β* influences neo-immunogenicity only marginally. Once *α* is further decreased, *β* influences neo-immunogenicity in a non-linear manner. Similar behavior can be seen for the non-junction cleavage likelihood. It decreases linearly with *α* and non-linearly with *β*. (PDF 51 kb) Additional file 3:
**Detailed results for comparing epitope pairs with and without spacers.** Detailed results for the comparison of epitope pairs with spacers and without spacers including sequences of the paired epitopes and designed spacers, predicted cleavage likelihoods of the two induced cleavage sites, and the combined cleavage likelihood, as well as the neo-immunogenicity of the epitope pair–spacer construct. (XLS 1236 kb) Additional file 4:
**Detailed results for comparing string-of-beads vaccines of different lengths with and without spacers.** Detailed results for the comparison of string-of-beads with spacers and without spacers including the string-of-beads sequences, predicted cleavage likelihoods, neo-immunogenicity, number of neo-epitopes, as well as the recovery rate of the desired epitopes. (XLS 299 kb) Additional file 5:
**Detailed prediction results for the polypeptide proposed by Levy et al.** Detailed results of the neo-epitope and cleavage site prediction analysis performed with PCM for cleavage site prediction, and with SYFPEITHI, BIMAS, and SMM for neo-epitope prediction for the polypeptide of Levy et al. (XLS 76 kb) Additional file 6:
**Comparison of different epitope prediction methods for in silico spacer design based on the polypeptide proposed by Levy et al.** Spacer sequences were constructed with SYFPEITHI, BIMAS, and SMM. Cleavage prediction was performed with PCM, classifying a site as cleaved if its score was greater than zero. The epitope thresholds used for neo-epitope detection were SYFPETHI-score ≥ 20, BIMAS ≥ 100 *T*_1/2_, and SMM ≤ 500 nM. *Red bars* represent predicted epitopes and the intensity indicates overlapping epitopes at that position. The *blue rectangles* represent predicted C-terminal cleavage sites. Spacer sequences are marked in *red*. A *tick* indicates the start position of a predicted nine-mer epitope. Although, the different prediction methods yielded different spacer sequences, the overall result remained the same. The in silico designed spacers were superior in terms of recovered epitopes and neo-epitope formation. (PDF 1198 kb) Additional file 7:
**Comparison of experimentally used and in silico designed spacers based on the polypeptide proposed by Ding et al.** *Red bars* represent predicted epitopes and the intensity indicates overlapping epitopes at that position. The *blue rectangles* represent predicted C-terminal cleavage sites. Spacer sequences are marked in *red*. A *tick* indicates the start position of a predicted nine-mer epitope. Epitope and cleavage site prediction were performed with SYFPEITHI and PCM, respectively. A nine-mer was predicted as an epitope if its predicted score was equal to or above a threshold of 20 (default threshold of SYFPEITHI). A cleavage site was said to be cleaved if the predicted PCM score was above zero. An epitope was defined as recovered if both the preceding and succeeding cleavage sites were predicted to be cleaved. (PDF 581 kb) Additional file 8:
**Detailed prediction results of the polypeptide proposed by Ding et al. **Detailed results of the neo-epitope and cleavage site prediction analysis performed with SYFPEITHI and PCM on the polypeptide of Ding et al. (XLS 43 kb)

## Abbreviations

EV: epitope-based vaccine
HLA: human leucocyte antigen
ILP: integer linear program
LO: lexicographical ordered optimization
PSSM: position-specific scoring matrix
SBV: string-of-beads
TSP: traveling salesperson problem
