# Supplementary material for: Designing string-of-beads vaccines with optimal spacers
Source: Genome Med. 2016 Jan 26;8:9. doi: 10.1186/s13073-016-0263-6 (PMC4728757; doi:10.1186/s13073-016-0263-6)
Supplement: Additional file 1: — Algorithm for string-of-beads design with flexible spacer sequences. A description in pseudo-code of the algorithm to determine the optimal ordering of epitopes and spacers for a string-of-beads vaccine. (PDF 1056 kb) [file 13073_2016_263_MOESM1_ESM.pdf]

String-of-Beads design with spacers of flexible length

Algorithm 1: Pseudocode for string-of-beads vaccines with spacer sequences of flexible length.

```
String-of-Beads with Spacer:
GIVEN:
  E: set of epitopes
  K: max. spacer length

INITIALIZE:
  A ← −∞        (adjacency matrix of graph G(E, E×E, A) )
  S ← ""         (spacer sequence for each pair of E×E)
  SBV ← ""      (final string-of-beads)

FOREACH e_i, e_j ∈ E×E DO:
  FOR k IN [0, K] DO:
    s_ij ← LO_spacer(e_i, e_j, k)
    IF min(C(e_i|s_ij), C(e_j|s_ij)) > A[i, j] DO:
      A[i, j] ← min(C(e_i|s_ij), C(e_j|s_ij))
      S[i, j] ← s_ij
    END IF
  END FOR
END FOREACH

SBV ← TSP_order(A, S)
RETURN SBV
```

Note: the two FOR-loops are parallelized.
