# Supplementary figures and images for: Designing string-of-beads vaccines with optimal spacers
Source: Genome Med. 2016 Jan 26;8:9. doi: 10.1186/s13073-016-0263-6 (PMC4728757; doi:10.1186/s13073-016-0263-6)

# Influence of $\alpha$ and $\beta$ with fixed spacer length k=3

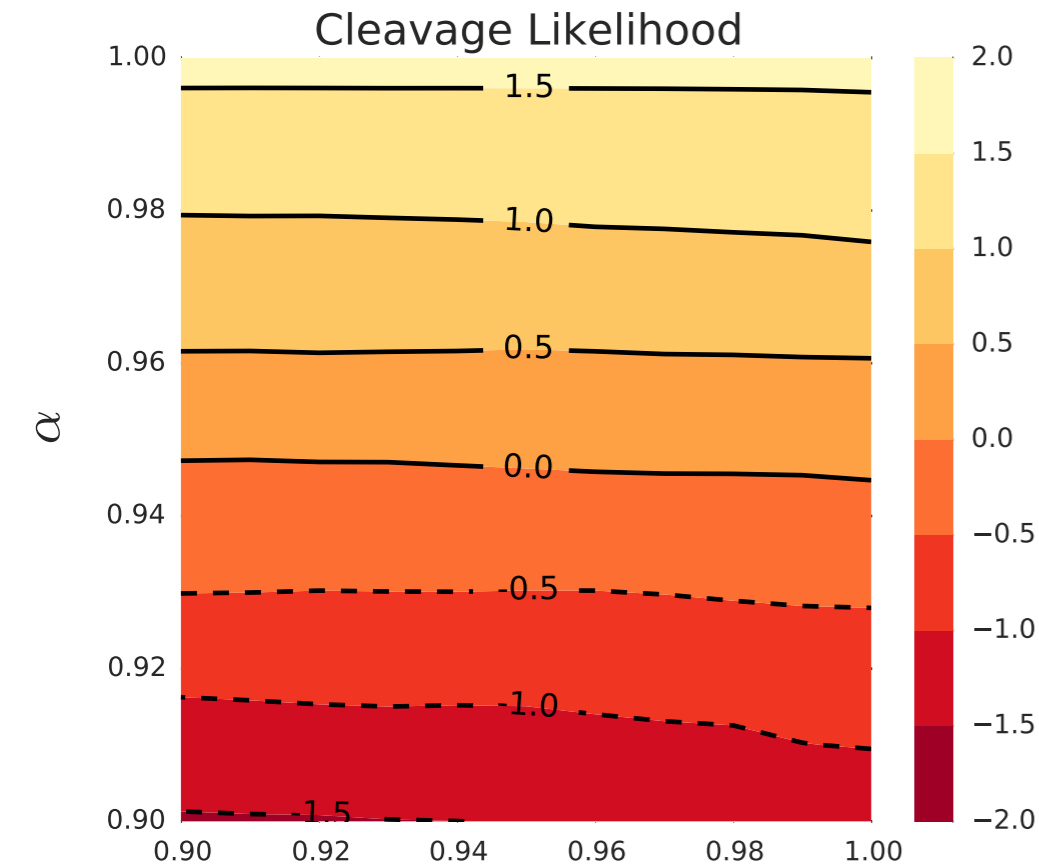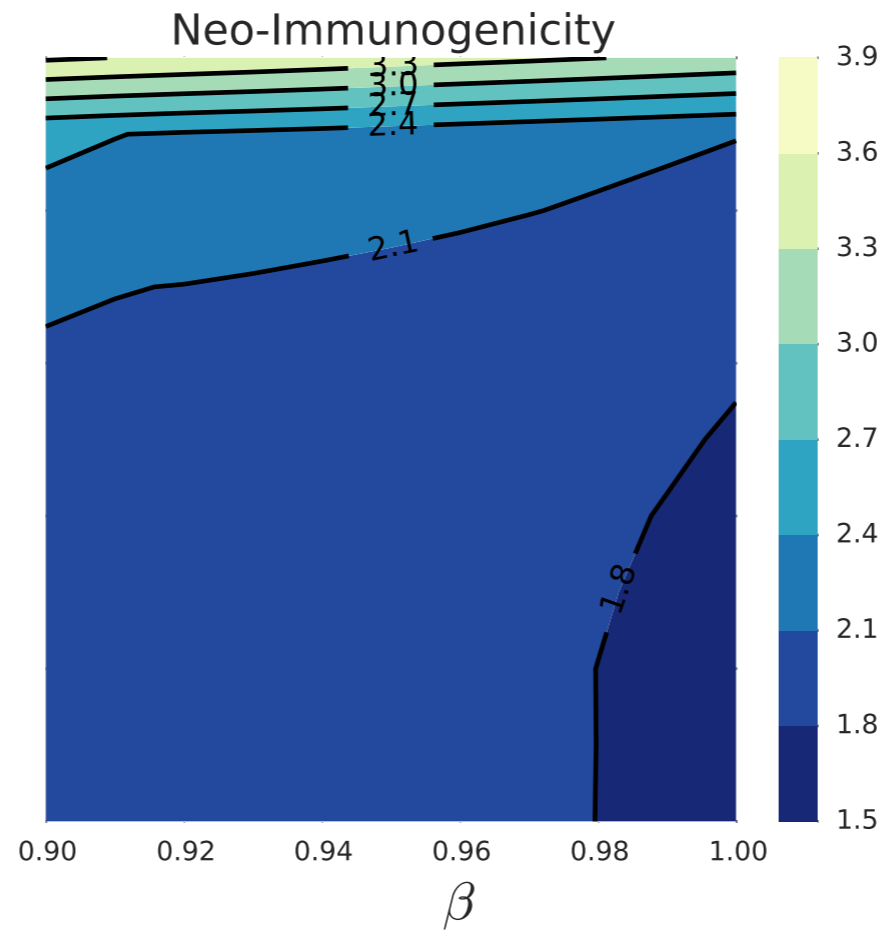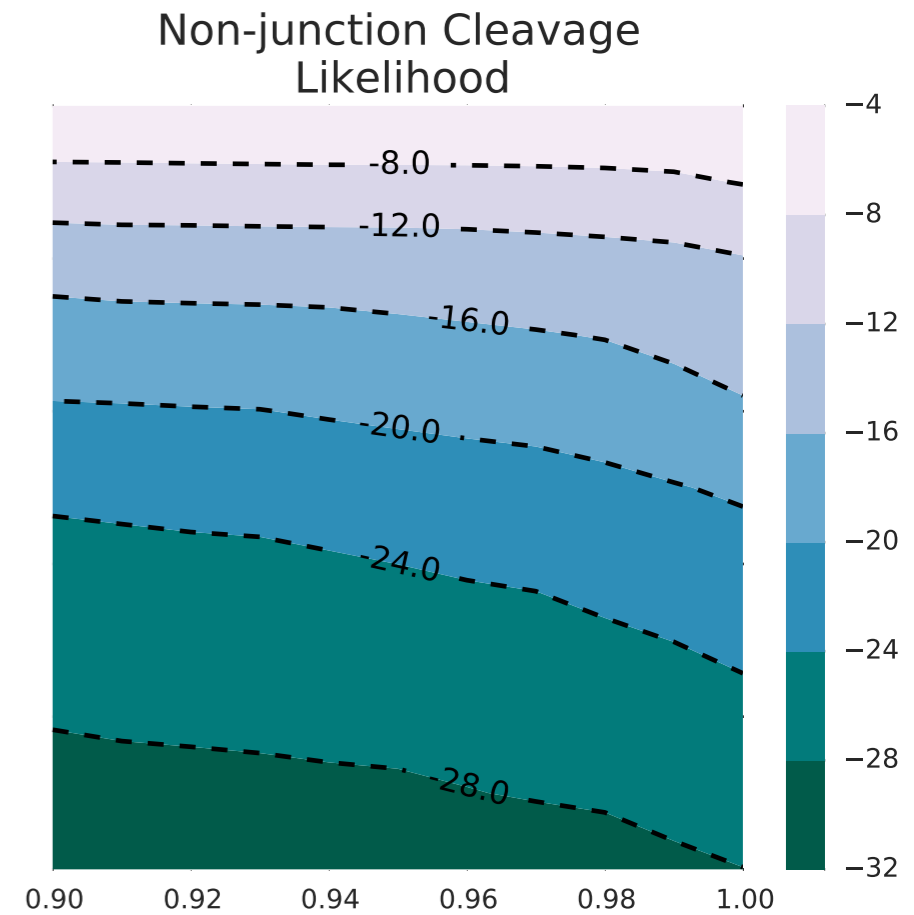

Supplement: Additional file 2: — Influence of α and β on cleavage likelihood, neo-immunogenicity, and non-junction cleavage likelihood exemplified for spacers of length three. Cleavage likelihood and neo-immunogenicity decrease linearly with α. For the conservatively chosen α = 0.99, β influences neo-immunogenicity only marginally. Once α is further decreased, β influences neo-immunogenicity in a non-linear manner. Similar behavior can be seen for the non-junction cleavage likelihood. It decreases linearly with α and non-linearly with β. (PDF 51 kb) [file 13073_2016_263_MOESM2_ESM.pdf]

SYFPEITHI

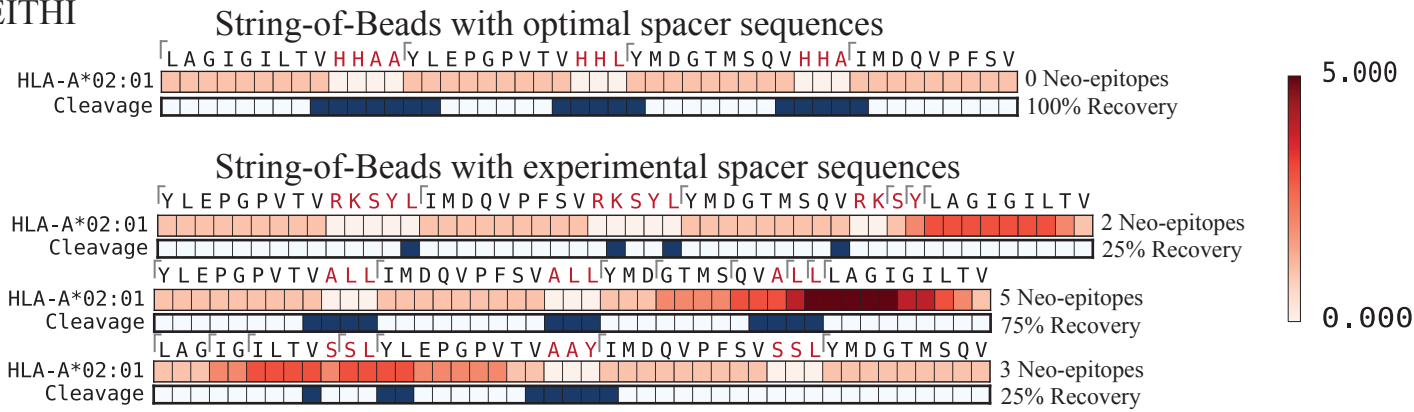

BIMAS

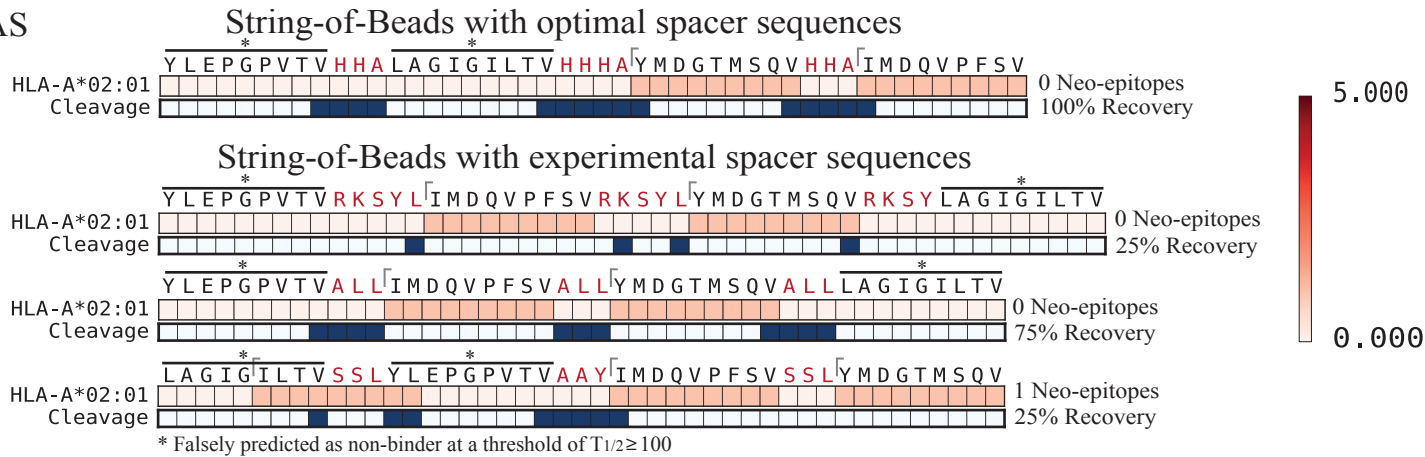

SMM

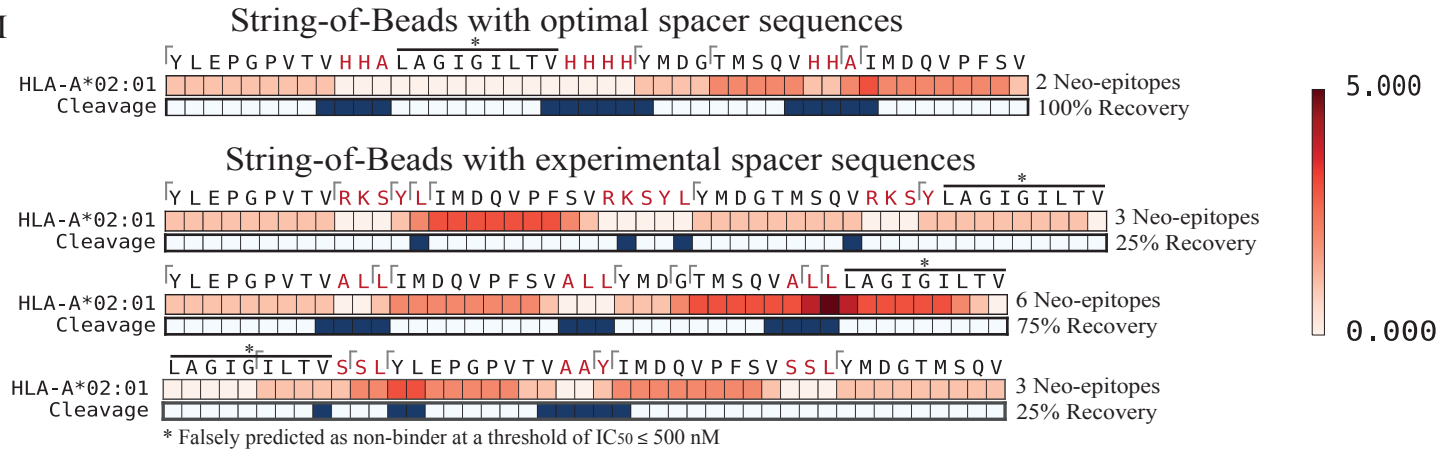

SYFPEITHI

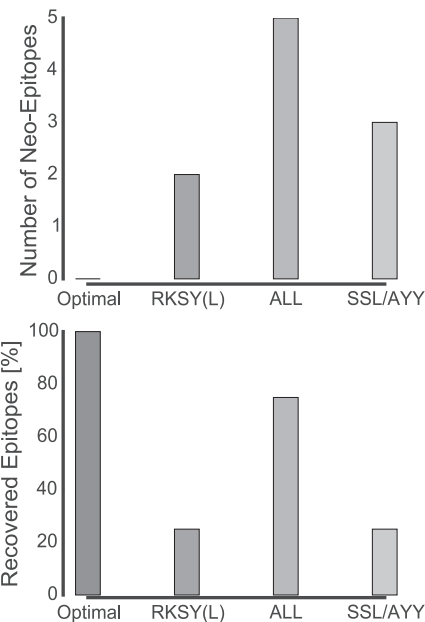

BIMAS

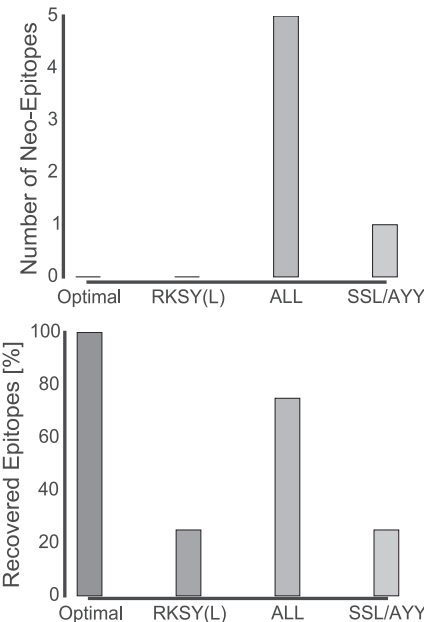

SMM

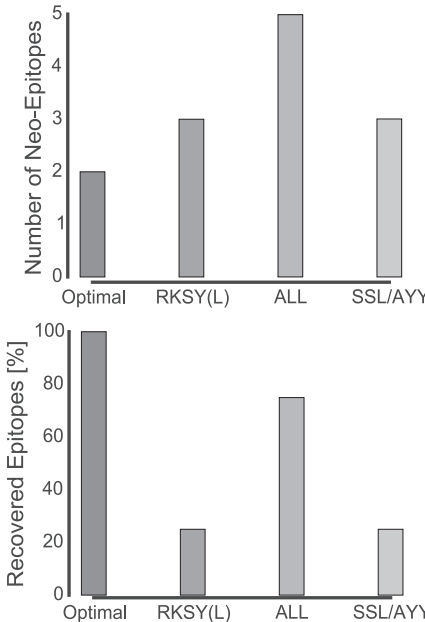

Supplement: Additional file 6: — Comparison of different epitope prediction methods for in silico spacer design based on the polypeptide proposed by Levy et al. Spacer sequences were constructed with SYFPEITHI, BIMAS, and SMM. Cleavage prediction was performed with PCM, classifying a site as cleaved if its score was greater than zero. The epitope thresholds used for neo-epitope detection were SYFPETHI-score ≥ 20, BIMAS ≥ 100 T 1/2, and SMM ≤ 500 nM. Red bars represent predicted epitopes and the intensity indicates overlapping epitopes at that position. The blue rectangles represent predicted C-terminal cleavage sites. Spacer sequences are marked in red. A tick indicates the start position of a predicted nine-mer epitope. Although, the different prediction methods yielded different spacer sequences, the overall result remained the same. The in silico designed spacers were superior in terms of recovered epitopes and neo-epitope formation. (PDF 1198 kb) [file 13073_2016_263_MOESM6_ESM.pdf]
