# Supplementary material for: Designing string-of-beads vaccines with optimal spacers
Source: Genome Med. 2016 Jan 26;8:9. doi: 10.1186/s13073-016-0263-6 (PMC4728757; doi:10.1186/s13073-016-0263-6)
Supplement: Additional file 7: — Comparison of experimentally used and in silico designed spacers based on the polypeptide proposed by Ding et al. Red bars represent predicted epitopes and the intensity indicates overlapping epitopes at that position. The blue rectangles represent predicted C-terminal cleavage sites. Spacer sequences are marked in red. A tick indicates the start position of a predicted nine-mer epitope. Epitope and cleavage site prediction were performed with SYFPEITHI and PCM, respectively. A nine-mer was predicted as an epitope if its predicted score was equal to or above a threshold of 20 (default threshold of SYFPEITHI). A cleavage site was said to be cleaved if the predicted PCM score was above zero. An epitope was defined as recovered if both the preceding and succeeding cleavage sites were predicted to be cleaved. (PDF 581 kb) [file 13073_2016_263_MOESM7_ESM.pdf]

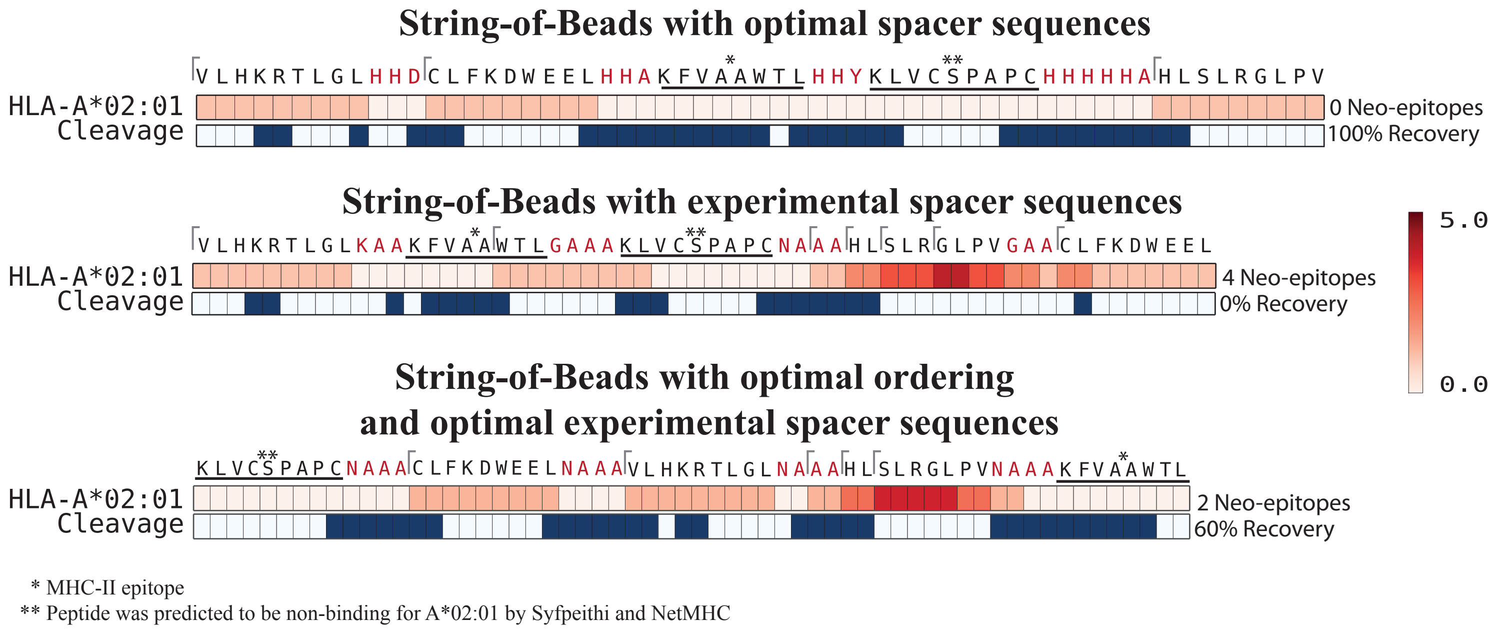
String-of-Beads with optimal spacer sequences
V L H K R T L G L H H D C L F K D W E E L H H A K F V A A W T L H H Y K L V C S P A P C H H H H H A H L S L R G L P V
HLA-A*02:01
Cleavage
0 Neo-epitopes
100% Recovery
String-of-Beads with experimental spacer sequences
V L H K R T L G L K A A K F V A A W T L G A A A K L V C S P A P C N A A A H L S L R G L P V G A A C L F K D W E E L
HLA-A*02:01
Cleavage
4 Neo-epitopes
0% Recovery
5.0
0.0
String-of-Beads with optimal ordering and optimal experimental spacer sequences
K L V C S P A P C N A A A C L F K D W E E L N A A A V L H K R T L G L N A A A H L S L R G L P V N A A A K F V A A W T L
HLA-A*02:01
Cleavage
2 Neo-epitopes
60% Recovery
* MHC-II epitope
** Peptide was predicted to be non-binding for A*02:01 by Syfpeithi and NetMHC
